# AI-generated documentation of psychiatric interviews: a proof-of-concept study

**DOI:** 10.3389/fpsyt.2026.1621532

**Published:** 2026-02-11

**Authors:** Bengican Gülegen, Raoul Haaf, Emanuel Schlüßler, Stephan Köhler

**Affiliations:** 1 Department of Psychiatry and Psychotherapy, Charité – Universitätsmedizin Berlin, Berlin, Germany; 2 Amnexis Digital Solutions GmbH, Berlin, Germany; 3 Alexianer St Joseph Berlin-Weißensee GmbH, Berlin, Germany

**Keywords:** artificial intelligence, clinical documentation, electronic medical records, natural language processing, neural language models

## Abstract

**Background:**

The documentation process in psychiatric interviews is laborious and often compromises the quality of patient care. Addressing this challenge, we explored the potential of artificial intelligence (AI) to automate documentation tasks and improve efficiency in psychiatric practice.

**Methods:**

Six simulated psychiatric interviews were transcribed and summarized using an AI model and compared to a gold standard, together with reports written by humans. Reports were decomposed into binary items using a predefined codebook covering patient information, current complaints, psychiatric history, medical history, medication, substance use, social history, family history, vegetative symptoms, psychopathology, and preliminary diagnoses. Transcription accuracy, performance, and inter-rater reliability were evaluated.

**Results:**

The AI achieved a high transcription accuracy with a mean word error rate of 9.44% and a Levenshtein score of 0.996, aligning with current voice-to-text transcription standards. Inter-rater reliability was high overall. The mean Cohen’s κ was 0.80 (SD = 0.33), the mean percent agreement was 0.96 (SD = 0.07), and the mean Gwet’s AC1 was 0.93 (SD = 0.12). Across all categories, human reports showed substantially higher agreement with the gold standard than AI reports. The mean accuracy was 0.94 (SD = 0.01) for human reports and 0.78 (SD = 0.08) for AI reports, t(5) = 6.33, p = .003. The mean F1 scores were also higher for human reports (M = 0.89, SD = 0.02) than for AI reports (M = 0.55, SD = 0.13), t(5) = 7.38, p = .001. Occasionally, AI reports provided more detailed contextual information than human reports. However, AI reports also introduced clinically relevant inaccuracies and struggled in complex domains such as psychopathology.

**Conclusions:**

While our findings suggest promising prospects for AI-driven documentation in psychiatry, further development is essential to enhance the model’s ability to comprehensively assess and document psychopathological features. Importantly, some AI-generated inaccuracies were clinically significant, underscoring the necessity of a final clinical review by a qualified professional. These findings are limited by the very small number of highly controlled simulated interviews. Larger studies with real patients, diverse clinicians, and routine clinical workflows will be required. Nonetheless, AI-supported documentation has the potential to considerably reduce time demands and alleviate the documentation burden in psychiatric care.

## 1 Introduction

### 1.1 Documentation burden

Documentation burden poses a particularly significant challenge across various domains of medicine, exerting high pressure on healthcare providers and impacting the delivery of quality patient care (1). In clinical settings, precise documentation serves as a cornerstone for effective communication, continuity of care, and legal compliance (2). However, the process of capturing and recording patient information is often plagued by its labor-intensive and time-demanding nature. This burden manifests in diverse ways, from the need to manually write detailed clinical encounters to the exhausting task of navigating complex electronic health record systems. As such, physicians devote an estimated one-third to two-thirds of their workday on electronic health records, having to spend more time on health records than on direct patient contact (3). For example, a study regarding the documentation burden for physicians in the USA showed a mean of 1.77 (95% CI, 1.67–1.87) hours daily spent on documentation outside of office hours (1). Another US-based study reported 59% of the on average 1.5 hours after work spent on electronic healthcare record (EHR) (3). During office hours, 49.2% of physicians’ total time was spent on EHR and desk work. That, in turn, can be directed to clinical burnout (4–6) and lack of documentation quality, which can even lead to clinical errors (7).

While documentation burden can be pervasive throughout all fields of medicine, its repercussions may be particularly pronounced within the field of psychiatry. Unlike many other medical specialties, psychiatry revolves around the nuanced exploration of patients’ mental health and emotional well-being, relying heavily on interpersonal communication and rapport-building during interviews (8). Additionally, legal requirements for documentation play a growing role in psychiatry (9). As such, the documentation process in psychiatry is not merely a logistical hurdle but a crucial component of holistic patient care, underscoring the need for innovative solutions to streamline and enhance documentation practices in psychiatry and psychotherapy.

### 1.2 AI solutions

Artificial intelligence (AI) presents promising solutions for addressing the documentation burden pervasive in psychiatric practice and medicine at large. With the power of machine learning algorithms and natural language processing techniques, AI technologies offer the potential to optimize clinical documentation workflows. Through automated transcription, summarization, and analysis of clinical encounters, AI systems may alleviate the manual burden of documentation, freeing up clinicians’ time for more meaningful patient interactions and therapeutic interventions. Moreover, AI-driven documentation solutions have the capacity to enhance the accuracy, completeness, and consistency of patient records, mitigating the risks associated with human error and variability in documentation practices (10).

Lin et al. (2018) presented a theoretical prototype for an AI recording patient–physician encounters via speech recognition and summarizing, sorting, and assembling clinical information. The authors went even further, suggesting the AI’s ability to make clinical recommendations, predict clinical risks, calculate scores, and conclude ICD codes (11). They concluded that many technologies necessary for such an autoscribe already exist. However, they presumed that it remains unclear how autoscribes can be exactly developed regarding the high amount of data necessary. In the meantime, some AI tools have been evaluated in other fields of medicine. For example, a proof-of-concept study proved the ability of a software tool to automatically create surgical reports (12). In the field of radiology, several products have been discussed to improve clinical documentation via AI, for example, through voice-recording examination rooms or smart watches that transcribe conversations and create clinical notes (10). However, there have been no AI solutions proposed for the psychiatric field.

### 1.3 Aims and hypotheses

In this study, we aimed to evaluate the functionality and potential applicability of an AI software solution that is currently in development and has been utilized across various other medical specialties (13). While AI technologies have demonstrated promising results in automating clinical documentation tasks (14, 15), their utilization and effectiveness in psychiatry remain relatively unexplored. Recognizing the unique challenges and nuances of psychiatric practice, we aimed to assess the feasibility and performance of this AI software within the context of psychiatric interviews and their documentation. By conducting a proof-of-concept evaluation, we aimed to elaborate on the strengths and limitations of implementing AI-driven documentation solutions in psychiatric settings.

We aimed to examine how effective the AI software would be in voice-to-text transcription and summarization by calculating its error rate, and especially in how accurately content would be assigned to its respective category within the psychiatric report, by comparing AI reports to human-written reports. Our goal was to explore not only overall quality and accuracy but also differences regarding different categories of psychiatric reports, to, for example, individually test the capability of recognizing and interpreting psychopathology as a key aspect of psychiatric evaluation.

## 2 Methods

### 2.1 AI software

We utilized an AI software called QUIXXS, which is currently under development by Amnexis Digital Solutions GmbH, an Ireland-based tech company specializing in digital health solutions utilizing AI (13). The software is already in use in several clinical fields, but has not yet been evaluated in psychiatric environments and has not been tested in clinical studies. The AI software runs on smartphones as an Android or IOS application and utilizes the recording function of the smartphone.

**Figure 1:**
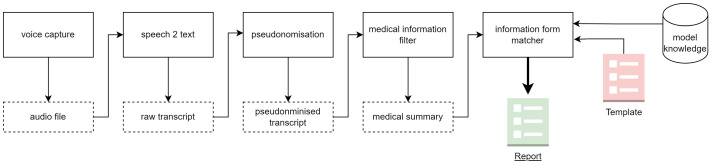
End to end processing pipeline for transforming recorded clinical speech into a structured medical report. Audio input is captured and transcribed, pseudonymised to protect patient identity, filtered to extract medically relevant content, and summarised. The resulting information is then matched against predefined templates and model knowledge to generate a standardised clinical report.

During a doctor–patient encounter, the smartphone is positioned in such a way that both the patient and the doctor can be heard well. The application can also be used for dictations without patients. The recorded audio data can be converted into a transcript using speech-to-text functions. To meet high data protection requirements, transcripts are initially pseudonymized. Afterward, transcription is performed using the Web API Whisper, a neural net automatic speech recognition (ASR) system by OpenAI, which has been proven to show human-like accuracy and robustness (16). Employing GPT-4, a large language model (17), a medical information filter compresses the pseudonymized transcript by extracting medically relevant information. A stream-based approach was employed, in which the transcript is divided into segments of approximately 5 minutes each. Every segment is compressed using a domain-specific summarization prompt. The resulting partial summaries are then sequentially linked together into an overall synthesis, which serves as the essential basis for form filling and further AI functions (e.g., cross-sectional analyses). In the final step, a previously selected form template is completed with the obtained information. Templates are used to specify the external form, structure, and desired content of a report. A data/form matcher fills the individual fields of the template with the medical information, thus completing the creation process. **Figure 1** depicts the processing pipeline of QUIXXS from audio input to clinical report generation.

### 2.2 Interview mode

For this proof-of-concept study, we simulated psychiatric interviews, as they would occur during the admission of patients for inpatient treatment at the clinic for psychiatry and psychotherapy of the University Hospital Charité Berlin Mitte. We conducted six interviews, one of which was held by a final-year medical student who was just completing their psychiatric rotation and conducting psychiatric interviews on the acute ward regularly, and two by a psychiatric resident. Interview partners, simulating a patient, were psychiatric nurses, residents, and volunteers who were not of a medical profession whatsoever. Those who were simulating to be a patient were instructed to present themselves as patients looking for psychiatric help because of some, not further specified, psychiatric symptoms. Those who were conducting the interview did not know beforehand which diagnosis the patients would introduce themselves with.

The interviews were held in the same manner as during a real, first physician–patient encounter on our acute psychiatric ward. Interviews were held in German and in accordance with standardized internal guidelines and protocols of our clinic, which were designed for psychiatric assessments. Patients were asked about their main symptoms, their psychiatric and medical history, their sociobiography, their family’s psychiatric history, and their current and recent medication. Psychopathology was assessed using a semi-structured interview according to the AMDP System: Manual for Assessment and Documentation of Psychopathology in Psychiatry (18).

During the interview, audio was recorded and then processed using QUIXXS. QUIXXS then interpreted from the recorded and transcribed audio files the role of the physician and the patient. Based on the transcribed and processed documents, QUIXXS assigned information to predetermined categories, such as patient information (including their name, age, and gender), current complaints or reason for presentation, psychiatric history, medical history, allergies, medication, social history, family history, vegetative symptoms, psychopathology, and preliminary diagnosis. The reports created automatically by QUIXXS were then saved as html files. After the interview, the physician wrote a report with the same, above-mentioned categories, as for a real inpatient treatment. The human reports were saved as Word files for future comparison. All reports were written in German, but excerpts for this paper were always translated into English for better understandability.

### 2.3 Measures

To test the quality of the AI reports, we tested error-proneness for two different steps of the report creation: transcription and reporting.

To test the accuracy of the voice-to-text transcription, the word error rate (WER) was used, which is the ratio of errors in a transcript to the total words spoken, the most common way to report the quality of voice-to-text transcription (19). Errors in this case would be substitutions, deletions, or insertions. For comparison, a transcript was created by a human as a ground-truth transcript. Both the human and AI transcripts were normalized: punctuation was deleted, and uppercase letters were changed to lowercase letters. Additionally, following changes were made: abbreviations were written out, slang words (such as “nah” instead of “no”) and contractions (“can’t” instead “cannot”, and “gonna” instead of “going to”) were written out, filler words (such as “hmm” or “uhm”) were deleted, and non-lexical conversational sounds were replaced with their equivalent (e.g., “no” instead of “uh-uh”). Grammatical errors of non-native speakers were corrected. Word error rate was then calculated using python v3.12.3 (20) and speechmatics python client v1.14.8 (21). In addition to WER, the Levenshtein score was calculated, which measures the similarity between the human and AI transcripts based on the minimum number of single-word edits (insertions, deletions, or substitutions) required to transform one into the other (22). This score provides a normalized similarity metric between 0 and 1, with 1 indicating perfect similarity. The calculation was performed using the python-Levenshtein library (23).

To assess the accuracy and quality of the reports, a reference standard was defined using the original audio recordings of the clinical interviews. A structured codebook was developed to operationalize systematic comparisons between this gold standard, the manually written reports, and the AI-generated reports. The codebook was grounded in standard domains of psychiatric assessment and comprised the following sections: personal information, current complaints, psychiatric history, somatic history, allergies, medication, substance use, social history, family history, vegetative symptoms, psychopathology, and preliminary diagnoses. Several sections of the codebook used multiple-choice response formats that combined fixed answer options with an additional free-text field. Fixed options covered the most frequently occurring categories, for example, commonly prescribed medications such as sertraline or mirtazapine, while free-text entries allowed entries that were not represented among the predefined options. Other sections relied exclusively on fixed single-choice options. An example is the assessment of visual hallucinations, with predefined responses of present and not present. If a report did not address visual hallucinations at all, no option was selected, and the corresponding item remained blank. Each response option within the codebook was treated as an independent binary item. For instance, within the substance use domain, nicotine use, alcohol use, and cocaine use were coded as separate variables. This design enabled a fine-grained, item-level analysis and deliberately avoided reliance on global free-text similarity measures. The exported codebook data were processed programmatically, and all responses were converted into string-based representations. Multi-select fields were automatically decomposed into individual binary indicators, resulting in a one-hot encoded item matrix in which each response option corresponded to exactly one analyzable variable. For each case and each item, concordance was assessed between the gold standard and the manual report, as well as between the gold standard and the AI-generated report. Items documented in a report but not supported by the audio recording were classified as false positives, whereas items present in the audio recording but absent from a report were classified as false negatives.

Structured codebook development and independent double coding, as employed here, follow recognized frameworks for quantitative content analysis in clinical research, providing a transparent and reproducible method for annotating and comparing key clinical concepts across sources.

### 2.4 Inter-rater reliability

To assess the inter-rater reliability of the proposed evaluation framework, a second independent rater re-rated all manual and AI-generated reports using the same codebook and rating procedure as the primary rater. Both raters were psychiatrically trained and blinded to each other’s ratings. Ratings were based on the structured item matrix derived from the original codebook, in which each answer option was represented as an individual binary item. Before statistical comparison, ratings were manually normalized to ensure semantic equivalence across raters, particularly for items derived from free-text inputs. This included harmonizing different phrasings referring to the same concept, for example, treating “leg fracture” and “broken leg” as equivalent. For each item, agreement between raters was computed on a per-case basis. Inter-rater reliability was primarily quantified using Cohen’s κ, which estimates agreement beyond chance for binary ratings (24). Because κ is sensitive to prevalence and marginal distributions, particularly in sparse or highly imbalanced items, two additional complementary measures were calculated. First, percent agreement was reported to provide an intuitive measure of absolute concordance (25). Second, Gwet’s AC1 was computed as a more robust chance-corrected agreement coefficient that is less affected by prevalence effects and is therefore recommended for reliability analyses involving binary clinical data with skewed distributions (26).

### 2.5 Statistical analyses

Performance was evaluated at both the global level and the section level. Accuracy was defined as the proportion of correctly classified items among all evaluated items. To account for class imbalance and to distinguish between overreporting and underreporting of clinical information, precision, recall, and the F1 score were additionally computed, with the F1 score defined as the harmonic mean of precision and recall. All performance metrics were calculated separately for manual reports relative to the gold standard and for AI-generated reports relative to the gold standard. In addition, absolute counts of false positive and false negative items were reported to characterize systematic error patterns. This evaluation framework, while novel in the psychiatric domain, follows established approaches for the objective assessment of medical AI outputs, in which performance is quantified using item-based accuracy, precision, recall, and F1 scores derived from false-positive and false-negative counts (27–29).

For performance comparison between manual and AI-generated reports, independent two-tailed t-tests were conducted. These analyses were used to test for differences in mean accuracy, precision, recall, and F1 scores between report types. All analyses and visualizations were performed using Python version 3.12.3 (30), and figures were generated using Matplotlib (31).

## 3 Results

### 3.1 Transcription

WER and Levenshtein scores are reported in **Table 1**. The mean WER was 9.44% (SD = 2.13%). Interviews contained an average of 2,684.33 words (SD = 800.68). Substitutions (M = 3.34%; SD = 1.60%) and deletions (M = 3.31%; SD = 1.21%) occurred more often than insertions (M = 2.77%; SD = 0.64%). The mean Levenshtein score was 0.966 (SD = 0.006).

**Table 1:** Report for the accuracy benchmarking for all three interviews, measuring the word error rate (WER) and Levenshtein score.

No.	Diagnosis	Native speaker	WER	Levenshtein score	Words (#)	Subst. (%)	Delet. (%)	Insert. (%)
1	DEP, SP, PD	No	0.08	0.9727	3,104	3.834	2.223	1.707
2	DEP, SP, PD, OCD	Yes	0.09	0.9637	2,325	3.484	2.968	2.538
3	DEP	Yes	0.12	0.9614	4,015	6.077	3.163	3.163
4	ADHD	Yes	0.11	0.9576	1,685	2.789	5.697	2.967
5	BPAD	Yes	0.07	0.9739	2,653	1.282	2.902	2.676
6	SCHIZ	Yes	0.09	0.9670	2,324	2.582	2.926	3.571

WER consists of substitutions (Subst.), deletions (Delet.), and insertions (Insert.). Diagnoses: depression (DEP), social phobia (SP), panic disorder (PD), obsessive–compulsive disorder (OCD), attention deficit hyperactivity disorder (ADHD), bipolar affective disorder (BPAD), and schizophrenia (SCHIZ).

However, some relevant errors occurred, which can be seen in **Table 2**. For example, when a patient was asked to spell the word “radio” backward to test for impairment of concentration, he was able to do so correctly. The AI transcript, however, only detected three of five letters correctly, therefore leading to a transcript that would suggest impairment of concentration, where there is none. Another example of significant mistakes was the misunderstanding of medical diseases. For instance, “hypertonia” was once mistaken for “hypothermia”, which could lead to a faulty medical history. Also, in many cases, names of medication were transcribed incorrectly, for example, “metazapine” instead of “mirtazapine” or “Zuprexa” instead of “Zyprexa”.

**Table 2:** Comparison of the AI transcript and the ground truth, with differences highlighted in bold.

AI transcript	Ground truth
Okay, could you spell the word radio backwards? Radio backwards? **O I D.** Okay great.	Okay, could you spell the word radio backwards? Radio backwards? **O I D A R**. Okay great.
My general practitioner prescribes me something against blood pressure. How is it called? It is called Ramipril, I think. Ramipril, okay, that means you have a known **hypothermia**?	My general practitioner prescribes me something against blood pressure. How is it called? It is called Ramipril, I think. Ramipril, okay, that means you have a known **hypertension**?
Now that I take the **Metazapine**, I sometimes feel a bit hungry again.	Now that I take the **Mirtazapine**, I sometimes feel a bit hungry again.
The medication was called **Zuprexa** I think.	The medication was called **Zyprexa** I think.

### 3.2 Reports

Inter-rater reliability was high overall. Across all 209 items and both report types combined, the mean Cohen’s κ was 0.80 (SD = 0.33), the mean percent agreement was 0.96 (SD = 0.07), and the mean Gwet’s AC1 was 0.93 (SD = 0.12), indicating robust agreement beyond chance despite heterogeneous item prevalence (mean prevalence = 0.24, SD = 0.21). For AI reports, the mean Cohen’s κ was κ = 0.75 (SD = 0.38), accompanied by a high mean percent agreement of 0.96 (SD = 0.09) and a mean Gwet’s AC1 of 0.93 (SD = 0.14), indicating robust agreement. For manually written reports, inter-rater reliability was similarly high, with a mean Cohen’s κ of 0.81 (SD = 0.36), a mean percent agreement of 0.96 (SD = 0.09), and a mean Gwet’s AC1 of 0.94 (SD = 0.15), reflecting slightly higher overall consistency between raters compared with AI-generated content.

**Figure 2:**
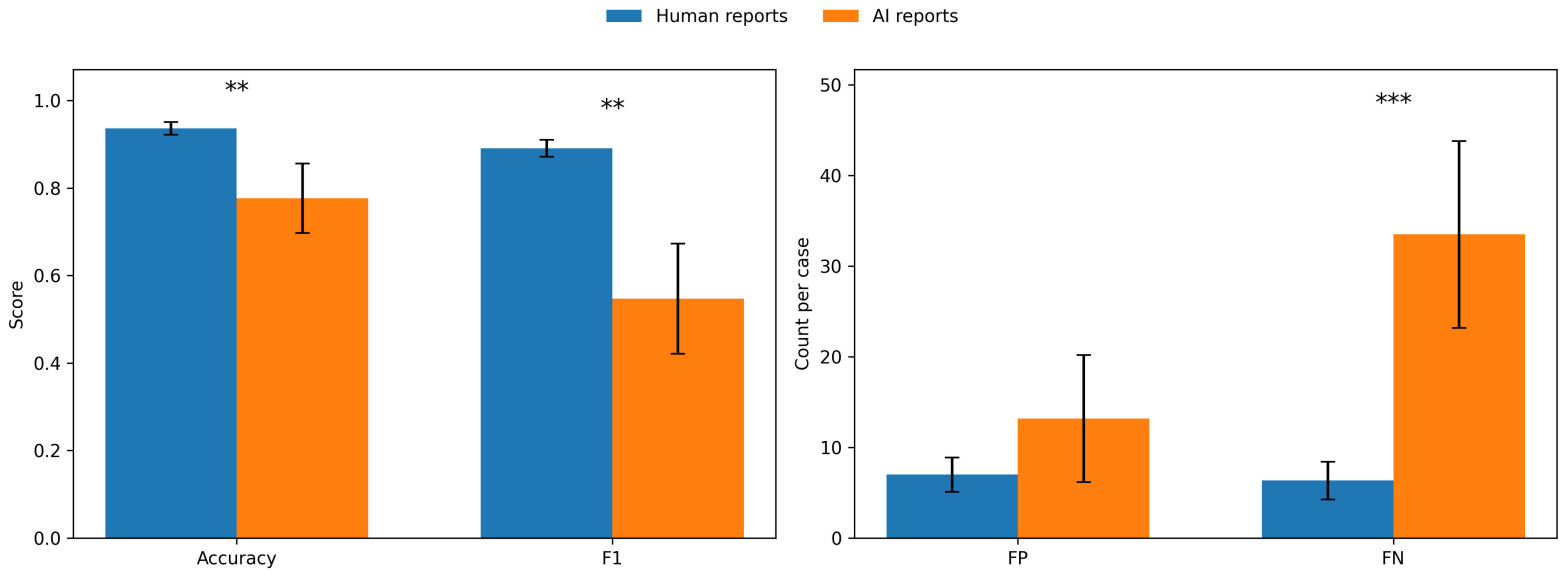
Global performance of human and AI-generated psychiatric reports compared to the gold standard. The left panel displays global accuracy and F1 scores, while the right panel shows the mean number of false positives (FP) and false negatives (FN) per case. Blue bars represent human-written reports, and orange bars represent AI-generated reports. Error bars indicate standard deviations across cases. Group differences between human and AI reports were tested using two-tailed paired t-tests. Statistically significant differences are marked with asterisks (**p <.01, and ***p <.001).

**Figure 2** shows accuracy, F1 scores, false negatives, and false positives for human and AI reports.

Across all categories and items, human-generated reports showed substantially higher agreement with the gold standard than AI-generated reports. Human reports achieved a mean accuracy of 0.94 (SD = 0.01), whereas AI reports reached a mean accuracy of 0.78 (SD = 0.08). This difference was statistically significant, t(5) = 6.33, p = .003. Similarly, mean F1 scores were significantly higher for human reports (M = 0.89, SD = 0.02) compared to AI reports (M = 0.55, SD = 0.13), t(5) = 7.38, p = .001. Error analysis revealed that AI reports produced more false negatives (M = 33.50, SD = 10.31) than human reports (M = 6.33, SD = 2.07), a difference that was statistically significant, t(5) = −8.39, p <.001. False positives did not differ significantly between human (M = 7.00, SD = 1.90) and AI reports (M = 13.17, SD = 7.03), t(5) = −2.02, p = .092.

**Figure 3:**
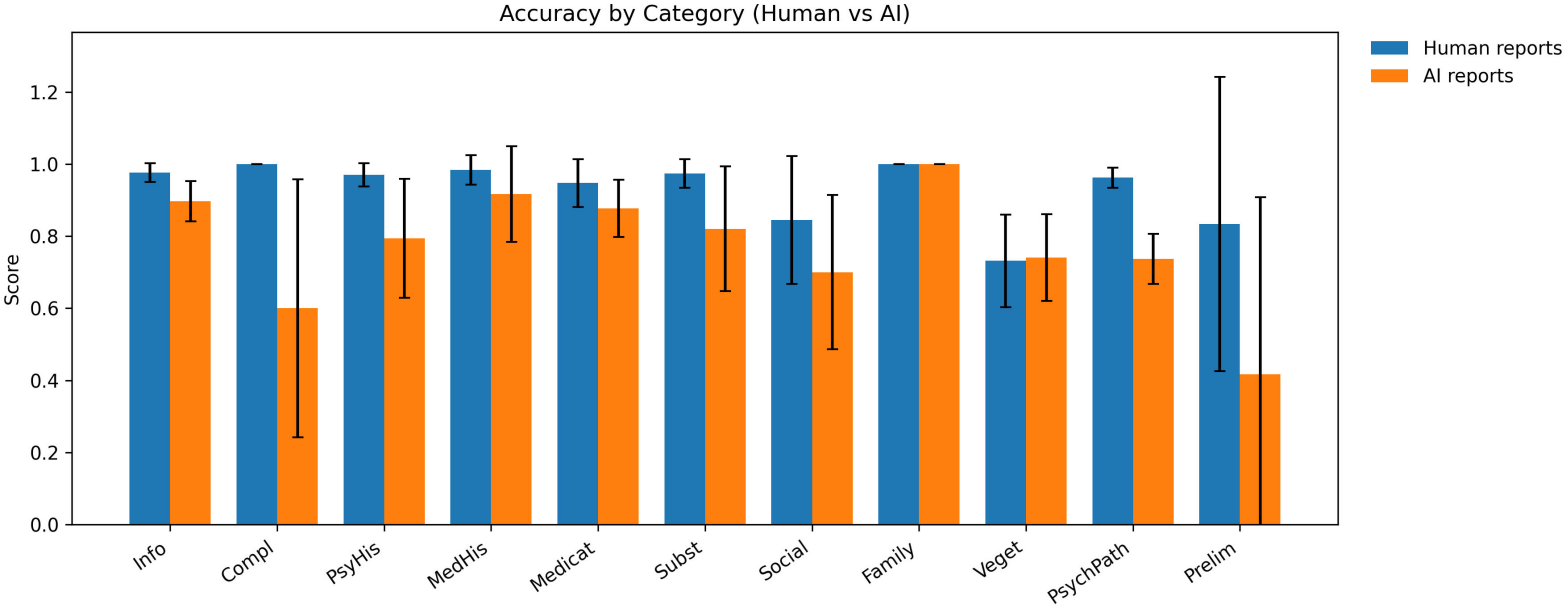
Accuracy by category for human and AI reports. The mean accuracy values for human-written and AI-generated psychiatric reports are shown separately for each diagnostic category, based on comparisons with the gold-standard annotations. Error bars represent standard deviations across cases. Accuracy reflects the proportion of correctly classified binary items within each category. Info, Patient Information; Compl, Current Complaints; PsyHist, Psychiatric History; MedHist, Medical History, Subst, Substance Use, Social, Social History, Family, Family History; Veget, Vegetative Symptoms; PsychPath, Psychopathology; Prelim, Preliminary Diagnosis.

**Figure 4:**
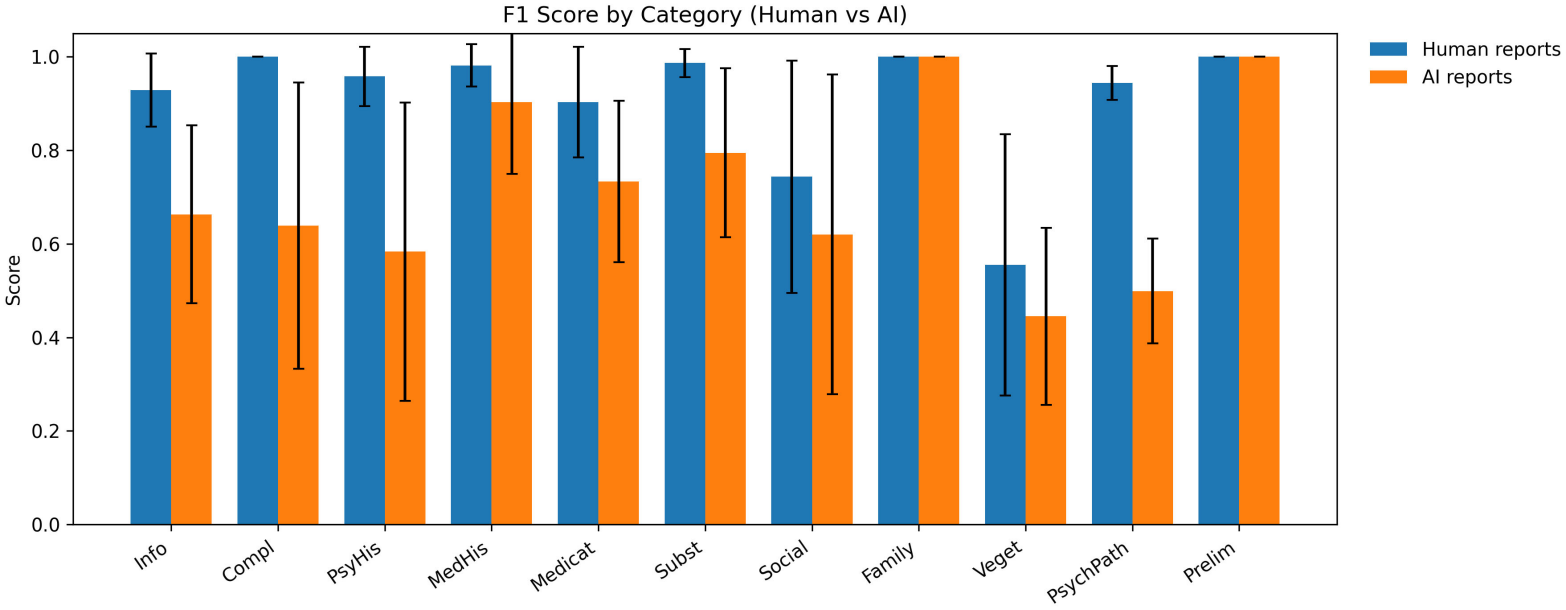
F1 scores by category for human and AI reports. The mean F1 scores for human-written and AI-generated psychiatric reports are shown separately for each diagnostic category, based on comparisons with the gold-standard annotations. Error bars represent standard deviations across cases. Accuracy reflects the harmonic mean of precision and recall relative to the gold standard. Info, Patient Information; Compl, Current Complaints; PsyHist, Psychiatric History; MedHist, Medical History, Subst, Substance Use; Social, Social History, Family, Family History, Veget, Vegetative Symptoms; PsychPath, Psychopathology; Prelim, Preliminary Diagnosis.

**Figure 3** shows the performance of AI reports throughout the different categories and **Figure 4** shows F1 scores. AI performance was the closest to human documentation in categories with limited semantic ambiguity and clearly defined factual content. For family history, AI achieved perfect agreement with the gold standard, with a mean accuracy of 1.00 (SD = 0.00) and an F1 score of 1.00 (SD = 0.00), matching human reports exactly (accuracy = 1.00, SD = 0.00; F1 = 1.00, SD = 0.00). Similarly, in medical history, AI reports demonstrated high accuracy (M = 0.92, SD = 0.13) and F1 scores (M = 0.90, SD = 0.15), approaching the performance of human reports (accuracy: M = 0.98, SD = 0.04; F1: M = 0.98, SD = 0.05). Comparable patterns were observed in medication history, where AI achieved an accuracy of 0.88 (SD = 0.08) and an F1 score of 0.73 (SD = 0.17), while human reports remained superior but closer in magnitude (accuracy: M = 0.95, SD = 0.07; F1: M = 0.90, SD = 0.12). For describing current complaints, AI performance declined more noticeably. AI reports reached a mean accuracy of 0.60 (SD = 0.36) and an F1 score of 0.64 (SD = 0.31), substantially below those of human reports, which achieved perfect performance in this category (both accuracy and F1: M = 1.00, SD = 0.00). This discrepancy reflects AI difficulties in consistently capturing symptom onset, course, and emphasis as expressed in free-form patient narratives. More pronounced performance gaps emerged for psychiatric history, where AI reports reached a mean accuracy of 0.79 (SD = 0.17) and an F1 score of 0.58 (SD = 0.32), compared to substantially higher values in human reports (accuracy: M = 0.97, SD = 0.03; F1: M = 0.96, SD = 0.06). Similarly, for psychopathology, AI performance was markedly lower, with a mean accuracy of 0.74 (SD = 0.07) and an F1 score of 0.50 (SD = 0.11), whereas human reports achieved near-ceiling performance (accuracy: M = 0.96, SD = 0.03; F1: M = 0.94, SD = 0.04). For social history, AI reports demonstrated moderate performance (accuracy: M = 0.70, SD = 0.21; F1: M = 0.62, SD = 0.34), again falling short of human documentation (accuracy: M = 0.84, SD = 0.18; F1: M = 0.74, SD = 0.25). Notably, both AI and human reports showed lower performance in vegetative symptoms, often omitting several items. AI achieved an accuracy of 0.74 (SD = 0.12) and an F1 score of 0.45 (SD = 0.19), which was similar in accuracy but lower in F1 compared to human reports (accuracy: M = 0.73, SD = 0.13; F1: M = 0.56, SD = 0.28). Finally, preliminary diagnoses revealed a distinctive pattern. While AI accuracy was low (M = 0.42, SD = 0.49), its F1 score reached 1.00 (SD = 0.00), mirroring human reports (accuracy: M = 0.83, SD = 0.41; F1 = 1.00, SD = 0.00). This reflects the binary structure of the diagnosis items and the fact that, when diagnoses were named, AI tended to reproduce them consistently, despite frequent omissions reflected in reduced accuracy.

**Table 3** shows examples of both human and AI reports, compared to the transcript, to visualize how information from the recorded conversation was converted to the report and to contextualize the quantitative performance differences across categories.

**Table 3:** Side-by-side comparison of the human and AI reports next to excerpts of the transcript.

Transcript	Human report	AI report
*Doctor:* Okay, and how long has this been going on? *Patient:* It has actually been like this for about two or three years, but in the past few weeks, it has gotten much worse again. (…) Then I basically spend the whole day either in bed or on the couch watching TV, but otherwise, I don’t do anything else anymore.	Current complaints (example of interview no. 1)	
	The symptoms have been present for 2–3 years but have significantly worsened in recent weeks. There is no specific trigger. The patient spends a lot of time in bed (…) and spends the rest of the day watching TV.	He has been feeling extremely exhausted and isolated for the past 2–3 years, and his condition has worsened in recent weeks. His daily routine mainly consists of lying in bed or on the couch and watching TV.
*Patient:* First, (my psychiatrist) prescribed me Sertraline. But that caused stomach problems for me, so he stopped it, and now I take Mirtazapine every evening. *Doctor:* Have there been any improvements? *Patient:* I might be able to fall asleep a bit better, but I still feel really bad. ∼ *Patient:* No, I’ve never had psychotherapy. (My psychiatrist) said I should search for a psychotherapist, but I just couldn’t manage it. ∼ *Patient:* And now with the Mirtazapine, I’ve maybe gained two kilos or so. ∼ *Doctor:* And have you ever had an inpatient stay? *Patient:* You mean psychiatric? No, I’ve never been inside of a psychiatric hospital.	Psychiatric history (example of interview no. 1)	
	His medication was switched from sertraline to mirtazapine due to gastrointestinal side effects. Under mirtazapine, there has been slight weight gain but improvement in sleep disturbances. No previous inpatient stays, no psychotherapy.	He previously took sertraline but stopped due to side effects. Psychotherapy has been recommended but has not yet been initiated.
*Doctor:* Do you take any other medication (…) besides the Mirtazapine? *Patient:* My general practitioner prescribes me something for blood pressure. *Doctor:* What is it called? *Patient:* It’s called Ramipril, I think. ∼ *Doctor:* But do you have anything else? *Patient:* I had my appendix removed once. *Doctor:* Okay. Do you have any allergies? You mentioned hay fever, but any allergies against medication? *Patient:* I’m allergic to penicillin.	Medical history and medication (example of interview no. 1)	
	Hypertension, appendectomy Allergies: penicillin and hay fever Medication: mirtazapine and ramipril	**n/c** Allergies: penicillin Medication: mirtazapine (for 1.5 years **against sleep problem**s) and ramipril (against hypertonia)
*Patient:* So, I’m actually studying. But I haven’t been able to attend my seminars in the last few months. And this semester, I haven’t managed to do anything. *Doctor:* What are you studying? *Patient:* I’m studying economics. ∼ *Doctor:* Do you live alone or in a shared apartment? *Patient:* I live in a shared apartment with another person, but they are also not home often. So, I am often alone. *Doctor:* So, you also feel lonely often? *Patient:* Yes, exactly. ∼ *Patient:* I have a few friends, but they don’t live nearby. So, I can’t see them very often. ∼ *Doctor:* And your family, where do they live? *Patient:* Um, they live in Braunschweig. (…) I talk to them on the phone now and then. ∼ *Doctor:* Do you have a partner? *Patient:* No, not anymore. *Doctor:* When did the relationship end, if I may ask? That was five or six **years** ago.	Social history (example of interview no. 2)	
	Studies economics, but could not continue for 4 months now. Had a job as a waitress, but had to quit due to a depressive episode. In Berlin, the patient has only sporadic contact with her friends. Her family lives in Braunschweig.	Lives in a shared apartment. Often feels lonely. Her family lives in Braunschweig, and they have contact by telephone. She has not had a partner for 5 to 6 years.

Errors are highlighted in bold.

An example of when AI performance was comparatively close to the human report and mirrored the interview content correctly is the current complaints section of interview no. 1. The patient reports a symptom duration of 2 to 3 years with marked worsening in recent weeks and describes spending most of the day in bed or on the couch watching television. Both the human report (“The symptoms have been present for 2–3 years but have significantly worsened in recent weeks…”) and the AI report (“He has been feeling extremely exhausted and isolated for the past 2–3 years…”) correctly captured symptom chronicity, functional impairment, and behavioral withdrawal.

In contrast, regarding their psychiatric history, in interview no. 1, the patient reports a switch from sertraline to mirtazapine due to gastrointestinal side effects, slight weight gain, improved sleep, absence of psychotherapy, and no prior inpatient treatment. While the human report explicitly integrated all these elements into a coherent longitudinal summary, the AI report selectively emphasized medication changes and side effects but omitted or fragmented contextual information, such as the lack of psychotherapy or inpatient stays. These omissions led to false negatives at the item level, reducing recall and F1 despite largely correct information.

In the medical history and medication categories, the excerpts show both the strengths and risks of AI enrichment. Compared with the human report, the AI sometimes appended additional medication context, such as an assumed indication or duration of treatment, even when the human report listed only the medication names. In interview no. 1, for example, the human report listed hypertension and appendectomy, penicillin and hay fever, and medication with mirtazapine and ramipril. The AI report, in contrast, included additional specifications, such as stating that mirtazapine had been prescribed for 1.5 years and assigning an indication (“against sleep problems”), and it also provided an indication for ramipril (“against hypertonia”). While indication and duration fields can be clinically useful, this excerpt also demonstrates the central failure mode: the assigned indication for mirtazapine was incorrect because the transcript indicates that mirtazapine was prescribed after sertraline for depressive symptoms, with sleep improvement reported as an effect rather than the primary reason for prescribing. This kind of plausible-sounding but incorrect attribute assignment would be expected to reduce precision and therefore depress F1, particularly in categories where correct attribution of diagnoses, indications, and historical treatments is central.

By contrast, social history errors more frequently involved abstraction and loss of specificity. In interview no. 2, the patient provides detailed information about studies, employment interruption, living situation, social isolation, geographic distance from friends, and family contact. The human report integrated these details into a structured narrative, whereas the AI report condensed the information into broader statements (“Often feels lonely” and “family contact per telephone”) and omitted temporal qualifiers such as the duration of unemployment or partnership status. These compressions are clinically plausible but failed to meet item-level criteria, leading to lower F1 scores despite superficially adequate summaries.

Sometimes, errors occurred due to faulty transcription. For example, once, the AI wrote that the patient had not been in a relationship for 5 to 6 years, although the patient stated that her relationship ended 5 to 6 *months* ago, missing a direct temporal link to the beginning of the current depressive episode, only because of the false transcription of *years* instead of *months*. In other cases, errors occurred, although the transcription was correct.

In some cases, the content of certain categories was misplaced altogether. **Table 4** shows a few examples where a category consisted of almost no adequate information. We could not identify what caused this error.

**Table 4:** Examples of unfitting or inadequate content within the AI reports.

AI report
Medical History (example of interview no. 5)
No obsessive–compulsive disorders, phobias, delusions, or hallucinations. Eating disorder in their youth? Previous physical illnesses? Hospital stays?
Vegetative symptoms (example of interview no. 5)
No further medication other than lithium and quetiapine? Allergies?
Psychiatric history (example of interview no. 4)
No anxiety, phobias, compulsions, panic attacks, hallucinations, or suicidal thoughts. No experience with a false reality, mind reading, secret messages, feeling persecuted, or malevolent people. No experience with unreal body sensations, loud thoughts, or foreign voices. Brief thoughts about non-existence, but no desire for suicide or self-harm. Mother has depression, and brother has ADHD without medication.

At last, we show examples of AI reports for psychopathology, the most challenging category. As can be seen in **Table 5**, in some cases, AI described certain aspects of psychopathology correctly, especially mood and drive, but sometimes also suicidal thoughts, compulsions, phobias, and panic attacks. Impairments in concentration or memory were correctly identified some of the time, but sometimes incorrectly as well. Both formal and content thought disorders were incorrect most of the time. Interestingly, one time, the human report was wrong, and the AI report was correct: in interview no. 2, the human incorrectly stated that the patient was able to recall three out of three words after 10 minutes, documenting no impairment of memory retention, although the ground-truth transcript proved that the patient did not memorize all three words, which was correctly stated by the AI and apparently incorrectly memorized or documented by the physician.

**Table 5:** Side-by-side comparison of the human and AI report.

Human report	AI report
Interview no. 1	
In contact: awake, conscious, friendly, approachable, and communicative. Fully oriented to place, situation, and person; temporal orientation not tested. Memory retention (1 out of 3 terms recalled after 10 minutes) and concentration reduced (0/5 in serial subtraction and 5/5 in backward spelling). No amnesia. Comprehension and abstract thinking not impaired. Worries about the future. Social phobia with avoidant behavior. Panic attacks and derealization experiences are present. No compulsive thoughts or actions. Formal thought is slightly slowed with a tendency to ruminate. No content thought disorders. No ego disturbances. Depressed mood with reduced emotional fluctuation. Severe lack of drive with a loss of everyday competencies. Psychomotor activity is normal. Thoughts of being tired of life with passive death wishes. No suicidal thoughts or plans. Credibly distanced from acute suicidality. No acute self- or other-directed danger. Insight into the illness is present.	(The patient) is awake and conscious. He is **oriented to time**, place, situation, and person. He **shows comprehension** and concentration disturbances, with **several errors in backward spelling** and serial subtraction. There are memory retention issues, as he could only remember a few terms after 10 minutes. There are indications of formal thought disorders such as ruminations and **thought pressure**. The patient exhibits **mistrust**, **hypochondria**, and **obsessive thoughts**. **There are content-related thought disturbances such as depersonalization and hallucinations.** The patient’s mood is depressed, and his affect is blunted. His drive is greatly reduced, and **psychomotor activity is slowed**. **Suicidality is present**. Psychovegetative abnormalities are present. **Insight into his condition is not clearly recognizable.**
Interview no. 2	
In contact: awake, conscious, friendly, approachable, and communicative. Slightly disoriented in time (Monday, January 9, 2024). Fully oriented to place, situation, and person. **Memory retention (3 out of 3 terms recalled after 10 minutes)** and concentration (5/5 in serial subtraction and 5/5 in backward spelling) objectively intact but subjectively reduced. Memory, comprehension, and abstraction in proverb testing are unimpaired. Fear of loneliness, social phobia, acrophobia, and claustrophobia with avoidance behavior. Panic attacks with fear of death. Depersonalization experiences. Compulsive actions (checking the stove and checking emails). No compulsive thoughts. Formal thought is slightly slowed with a tendency to ruminate. No content thought disorders. No perceptual disturbances. No ego disturbances. Depressed mood with reduced emotional fluctuation. Reduced drive. Psychomotor activity is normal. Suicidal thoughts and methods (medication overdose), but credibly distanced from acute suicidality. No acute self- or other-directed danger. Insight into the illness is present.	(The patient) is awake and conscious. She is **oriented to time**, place, situation, and person. **Comprehension and concentration disturbances in backward spelling and serial subtraction were noted**. She had memory retention issues, recalling only a few terms after 10 minutes. **Formal thought disorders such as thought pressure and flight of ideas were observed**. The patient shows fears of loneliness, heights, confined spaces, and social interactions. She suffers from panic attacks, compulsive actions, suicidal thoughts, identity loss, memory problems, sadness, lack of drive, and ruminations. The patient’s mood is depressed, and her affect is blunted. Her drive is reduced, and **psychomotor activity is slowed**. Suicidality is present. Psychovegetative abnormalities such as sleep disturbances, loss of appetite, and weight loss were found. **Insight into her condition is limited**.

Errors are highlighted in bold.

## 4 Limitations

This study has several limitations that should be acknowledged. First and most importantly, the work was based on simulated psychiatric interviews conducted under highly controlled conditions. Real-world clinical interviews show much greater variability in communication style, emotional tone, comorbidity, and the expression of psychopathology and often involve complex relational dynamics. It is therefore likely that model performance in actual clinical practice would be lower than in the present simulations. Future studies should therefore be conducted in diverse clinical environments to gather comprehensive data on how the AI performs across different psychiatric conditions and patient demographics.

Second, the sample size was extremely small, which means that all statistical analyses must be considered exploratory and that effect estimates were unstable. The results should thus be understood as preliminary signals that require confirmation in larger and more diverse samples of patients and clinicians.

Third, no systematic calibration analysis (e.g., through temperature scaling, Platt scaling, or reliability diagrams) was conducted. In the context of psychiatric interview transcription and summarization, mispredictions made with high confidence could be particularly problematic, as they may introduce clinically significant misunderstandings that remain unchecked. Our primary focus was on the qualitative evaluation of generated notes in collaboration with subject-matter experts, and calibration was therefore not included in the methodological scope. Future work should incorporate calibration assessments to better characterize and mitigate the risks of overconfident errors in clinical applications.

Finally, no automated metrics such as ROUGE or embedding-based similarity were applied for the evaluation of summarization quality. Psychiatric interview reports are inherently heterogeneous in structure, sequence, and focus, even between human clinicians, which reduces the interpretability and clinical relevance of such metrics. Small differences in phrasing or emphasis may result in low similarity scores despite equivalent clinical accuracy. To address this challenge, we instead adopted an approach with a structured codebook development and independent double coding, following recognized frameworks for quantitative content analysis.

Taken together, our findings should be viewed as an early feasibility and method study rather than as evidence for clinical implementation. The main contribution of this work is to demonstrate that it is technically possible to generate structured psychiatric documentation directly from recorded interviews and to outline a concrete evaluation framework that combines transcription accuracy with clinically meaningful content metrics. The study is not designed or powered to support strong claims about clinical effectiveness or safety, and it does not address how such a system would perform in the full heterogeneity of routine care.

## 5 Discussion

While acknowledging the above-stated limitations of this study, our results suggest a significant potential for AI to streamline the documentation process and reduce documentation burden for clinicians in psychiatry. However, our findings regarding erroneous and redundant information point to the need for refining the AI to reduce inaccuracies and superfluous content to ensure reliable documentation.

Specifically for psychiatry, there was one great weakness of the AI: Its performance in documenting psychopathology. This underscores a critical area for further development, as accurate and structured documentation of psychopathology is essential for psychiatric evaluations (32). Recognizing psychopathology can be quite a demanding task, with the need for professional training in the psychiatric field. There are several aspects of psychopathology that can only be assessed by professionals with enough experience and expertise, for instance, to identify and distinguish between delusions, partial delusions, and preoccupations. One solution would be further, specific training of the AI for psychopathology. In a future study, we plan to train the AI on a high number of psychopathology reports for it to learn the general structure and combine this with audio records or transcripts from the doctor–patient conversations from which the psychopathology reports were obtained (33). However, this can be a difficult task to accomplish, as audio records from real doctor–patient encounters in psychiatry are rare and highly sensitive and could sometimes even risk patients’ privacy (34). Another hurdle would be that some aspects of psychopathology are not exclusive to the content of what patients say, but the intonation and volume of their way of talking (35). Meanwhile, the approach of the AI model that we examined focuses only on written transcripts, giving no insight into the sound or nature of speech. Additionally, some aspects of psychopathology are not apparent through speech at all. For example, psychomotor disorders can only be able to be perceived visually (18). However, the AI guessed those disorders, eventually wrong in many cases. AI, therefore, should be trained to only describe those aspects of psychopathology that can accurately be described by the content of an interview. Alternatively, developers would have to find additional ways to assess changes in tonality and volume of speech, and even visual analysis via video.

However, the AI demonstrated a significant potential to reduce the documentation burden by automating substantial portions of the documentation process. This could free up clinicians’ time for direct patient care and other critical tasks, potentially improving the overall efficiency and quality of psychiatric care (10). Even with their current restrictions, we suppose that the AI would already significantly reduce the time spent on documentation, as even the brief correction of its mistakes would cost a lot less time than writing the whole report manually. To prove this hypothesis in future studies, we plan to include time measurements to compare the time spent on manual report creation and the time spent on revision and correction of AI reports. Only by obtaining exact time measurements will a quantitative assessment of the effectiveness of AI-driven scribes be possible, which is the key point in testing whether the documentation burden can be reduced.

Importantly, it is worth noting that some AI-generated inaccuracies were highly clinically relevant. For example, previous diagnoses were misunderstood, and the wrong names of medication were recorded. This underscores that human supervision remains a crucial component in ensuring the safety and accuracy of AI-generated documentation (36). Moreover, the involvement of clinicians in the review process can provide an additional layer of quality control and continuous improvement for the AI system. By systematically analyzing and correcting the AI’s errors, clinicians can help refine the algorithms and contribute to the development of more accurate and reliable AI tools (12). This iterative feedback loop is essential for enhancing the AI’s performance and ensuring that it evolves to meet the complex demands of real-world psychiatric practice.

However, integrating AI into clinical documentation processes raises important considerations about the clinician–patient relationship (37, 38). There is a potential risk that over-reliance on AI could depersonalize patient care, as clinicians may spend less time engaging directly with patients. To mitigate this, it is important to strike a balance where AI supports clinicians by reducing administrative burdens without diminishing the quality of interpersonal interactions and empathetic patient care. There is evidence that to ensure a positive impact on the clinician–patient relationship, AI should not *replace* but rather *assist* clinicians in treatment (37). Future studies should also explore the impact of AI integration on patient satisfaction and the therapeutic alliance, ensuring that technological advancements complement rather than compromise the human aspects of psychiatric practice.

The deployment of AI in the psychiatric field also raises important ethical and legal questions and ensures that patient confidentiality, data security, and adherence to legal standards will be of high importance (39–41). Under the European General Data Protection Regulation (GDPR), audio recordings of psychiatric consultations and their automated analysis constitute the processing of particularly sensitive personal data (42). Any clinical deployment, therefore, requires a clearly defined legal basis, for example, within the framework of medical treatment and quality assurance, as well as strict adherence to the principles of data minimization, purpose limitation, and storage limitation. In practice, this implies that patients must be transparently informed about the nature and purpose of the recordings, the role of the AI system, and the limits of its use (43). They should understand that the clinician remains fully responsible for the content of the final report and for all diagnostic and therapeutic decisions and that AI-generated content is only a draft that requires critical review (44). The technical implementation of AI-supported documentation must also meet high standards of data protection and information security (45, 46). Whenever possible, processing should occur within secure institutional infrastructures, and the use of external cloud services needs scrutiny, contractual safeguards, and data protection impact assessments. Current debates on AI-supported clinical documentation in psychiatry highlight concerns about potential misuse of sensitive data, unintended secondary uses, and the risk that automated summaries could be accessed by parties beyond the immediate treatment team (47, 48). These concerns underline the importance of governance structures that involve data protection officers, ethics committees, clinicians, and patient representatives. Our proof-of-concept study deliberately avoided many of these issues by relying on simulated interviews and by processing the data in a controlled research environment. Future work that moves toward real patient data will need to integrate ethical and legal considerations from the outset and to demonstrate not only technical performance but also compliance with data protection law and acceptance by patients and clinicians.

## 6 Conclusions

In conclusion, this proof-of-concept study suggests that AI systems can generate usable draft documentation from psychiatric interviews but also reveal important limitations. The AI achieved high transcription accuracy and produced structured reports that could be systematically evaluated. The evaluation methodology itself demonstrated high inter-rater reliability across both human and AI-generated reports, supporting the robustness of the coding and comparison procedure. While AI-generated reports showed substantially lower overall performance than human reports, it varied markedly across clinical domains. In more factual and demographically oriented categories, AI performance approached human-level agreement with the gold standard. Given the very small and simulated sample, these findings are preliminary and should be considered hypothesis-generating. The main contribution of this work is an evaluation framework and initial signal of feasibility, not evidence for clinical deployment. Future work in real-world settings, combined with robust safeguards for data protection and clinical oversight, will be essential. Nonetheless, AI-supported documentation has the potential to considerably reduce time demands and alleviate the documentation burden in psychiatric care.

## Data Availability

The raw data supporting the conclusions of this article will be made available by the authors, without undue reservation.
